# Deletion of Monoglyceride Lipase in Astrocytes Attenuates Lipopolysaccharide-induced Neuroinflammation*

**DOI:** 10.1074/jbc.M115.683615

**Published:** 2015-11-12

**Authors:** Gernot F. Grabner, Thomas O. Eichmann, Bernhard Wagner, Yuanqing Gao, Aitak Farzi, Ulrike Taschler, Franz P. W. Radner, Martina Schweiger, Achim Lass, Peter Holzer, Erwin Zinser, Matthias H. Tschöp, Chun-Xia Yi, Robert Zimmermann

**Affiliations:** From the ‡ Institute of Molecular Biosciences, University of Graz, 8010 Graz, Austria,; § the Institute of Biomedical Science, FH Joanneum University of Applied Sciences, 8020 Graz, Austria,; the ¶ Institute of Diabetes and Obesity, Helmholtz Center Munich, 85748 Garching, Germany,; the ‖ Institute of Experimental and Clinical Pharmacology, Medical University of Graz, 8010 Graz, Austria, and; the ** Department of Endocrinology and Metabolism, Academic Medical Center Amsterdam, 1105 Amsterdam, The Netherlands

**Keywords:** arachidonic acid (AA) (ARA), astrocyte, endocannabinoid, lipopolysaccharide (LPS), neuroinflammation

## Abstract

Monoglyceride lipase (MGL) is required for efficient hydrolysis of the endocannabinoid 2-arachidonoylglyerol (2-AG) in the brain generating arachidonic acid (AA) and glycerol. This metabolic function makes MGL an interesting target for the treatment of neuroinflammation, since 2-AG exhibits anti-inflammatory properties and AA is a precursor for pro-inflammatory prostaglandins. Astrocytes are an important source of AA and 2-AG, and highly express MGL. In the present study, we dissected the distinct contribution of MGL in astrocytes on brain 2-AG and AA metabolism by generating a mouse model with genetic deletion of MGL specifically in astrocytes (MKO^GFAP^). MKO^GFAP^ mice exhibit moderately increased 2-AG and reduced AA levels in brain. Minor accumulation of 2-AG in the brain of MKO^GFAP^ mice does not cause cannabinoid receptor desensitization as previously observed in mice globally lacking MGL. Importantly, MKO^GFAP^ mice exhibit reduced brain prostaglandin E2 and pro-inflammatory cytokine levels upon peripheral lipopolysaccharide (LPS) administration. These observations indicate that MGL-mediated degradation of 2-AG in astrocytes provides AA for prostaglandin synthesis promoting LPS-induced neuroinflammation. The beneficial effect of astrocyte-specific MGL-deficiency is not fully abrogated by the inverse cannabinoid receptor 1 agonist SR141716 (Rimonabant) suggesting that the anti-inflammatory effects are rather caused by reduced prostaglandin synthesis than by activation of cannabinoid receptors. In conclusion, our data demonstrate that MGL in astrocytes is an important regulator of 2-AG levels, AA availability, and neuroinflammation.

## Introduction

Monoglyceride lipase (MGL)^2^ is a serine hydrolase widely expressed throughout the CNS (1, 2) and is the major degradative enzyme for 2-arachidonoylglyerol (2-AG) in the brain (3). 2-AG is the most abundant endogenous agonist of cannabinoid receptors (CBR1/2) and is hydrolyzed by MGL to arachidonic acid (AA) and glycerol (1). CBRs have been originally identified as molecular targets of Δ-9-tetrahydrocannabinol, the psychoactive component of *Cannabis sativa* (4). Their endogenous agonists, including 2-AG, are therefore designated as endocannabinoids. Previous studies showed that global genetic deletion of MGL in mice (MKO^global^) leads to massively increased brain 2-AG levels, but does not provoke cannabimimetic behavioral effects (5, 6). This was explained by CBR desensitization leading to functional antagonism (5–7).

Published data suggest that MGL has a dual function. First, it controls the levels of 2-AG, which exhibits neuroprotective effects by preventing excitotoxicity in neurons (8, 9), but also by directly acting on microglia (10). Second, by degrading 2-AG, MGL provides AA for eicosanoid synthesis, a process which was originally thought to be exclusively dependent on phospholipase A2 (11). Accordingly, mice globally lacking MGL exhibit reduced brain AA and prostaglandin levels and are protected against LPS-induced neuroinflammation (12).

Astrocytes actively contribute to brain endocannabinoid signaling by secreting 2-AG (13) and by releasing gliotransmitters upon activation of CBRs (14). Furthermore, they are considered as key players in the synthesis and provision of polyunsaturated fatty acids in brain (15) and, together with microglia, they critically determine the inflammatory response of the CNS (16). Since MGL is highly expressed in astrocytes (13, 17), we dissected the distinct contribution of MGL in astrocytes on brain 2-AG levels and AA metabolism. For this purpose, we generated a mouse model with specific genetic deletion of MGL in astrocytes and examined brain 2-AG and AA metabolism, behavior, and neuroinflammation. Our data demonstrate that astrocyte-specific deletion of MGL increases brain 2-AG levels without causing CBR desensitization or cannabimimetic behavioral effects. Yet, MGL-deficiency in astrocytes reduces the availability of AA for prostaglandin synthesis and thereby attenuates LPS-induced neuroinflammation.

## Experimental Procedures

### 

#### 

##### Animals

Mice were maintained on a regular dark light cycle (12 h light, 12 h dark) at 22 ± 1 °C in a specific pathogen free (SPF) environment and were and kept *ad libitum* on a standard laboratory chow diet (M-Z extrudate, V1126, ssniff). MKO^GFAP^ mice were generated by targeted homologous recombination, similar as described for MKO^global^ mice (7). In brief, a targeting vector harboring *Mgll* exons 3 and 4 flanked by a floxed neomycin resistance gene cassette and an additional *loxP* site was used for homologous recombination in murine embryonic stem cells. Embryonic stem cell clones with a manipulated *Mgll* locus were then transfected with a plasmid encoding Cre-recombinase to obtain embryonic stem cell clones with a floxed *Mgll* allele. Two independent clones were selected for microinjection into 3.5-day-old C57BL/6J blastocysts followed by implantation into recipient mice. Chimeric animals were bred with C57BL/6J mice to obtain *Mgll* floxed mice. Astrocyte specific deletion was achieved by breeding *Mgll* floxed mice with mice transgenic for Cre-recombinase under control of the human GFAP-promotor (FVB-Tg(GFAP-cre)25Mes/J, The Jackson Laboratory). Subsequently, female mice homozygous for the floxed *Mgll* allele and heterozygous for the Cre-recombinase were bred with male mice homozygous for the floxed *Mgll* allele. *Mgll* floxed littermates were used as wild-type controls. To exclude effects of Cre-recombination *per se* (18) C57BL/6J mice carrying the Cre-recombinase under control of the human GFAP-promotor (WT ^GFAP^) (19, 20) were used as controls. No differences between wild-type and WT^GFAP^ mice were observed (data not shown). The study was approved by the Ethics committee of the University of Graz, the Austrian Federal Ministry of Science and Research, and is in accordance with the council of Europe Convention (ETS 123).

##### Primary Astrocyte Cultures

Brains of 1 day old pups were excised, meninges were removed, and brain tissue was washed in MEM alpha medium (Life Technologies, Thermo Scientific). Subsequently, brain tissue was mechanically triturated, filtered through a 70-μm cell strainer and centrifuged at 1,000 × *g* for 5 min. The cell pellet was resuspended in MEMα containing 10% FCS, 2 mm glutamine (Life Technologies, Thermo Scientific), 100 IU/ml penicillin/streptomycin (Gibco, Thermo Scientific), and 100 μg/ml Primocin (InvivoGen, San Diego, CA). Cells were seeded in flasks coated with 15 μg/ml poly-d-lysine (Merck KGaA) and cultivated under standard conditions at 37 °C, 5% CO_2_, and 95% humidified atmosphere. Microglial cells were mechanically detached and removed via changing the medium every 3 days.

##### Primary Microglia Cultures

Microglia were obtained from mixed astrocyte/microglia cultures. Therefore, astrocytes were isolated and cultivated as described, but no detachment of microglia was performed. 10 days after astrocyte cultures reached confluency, microglia were harvested by mechanical detachment and centrifugation at 1,000 × *g* for 5 min. Microglia were seeded in 15 μg/ml poly-d-lysine (Merck KGaA)-coated dishes and maintained at 37 °C, 5% CO_2_, and 95% humidified atmosphere.

##### Primary Neuron Cultures

Cortical and hippocampal neurons were isolated from day 15.5 embryonic brains after removal of meninges via dissection in papain solution (Worthington). Neurons were seeded on cultivation dishes coated with 50 μg/ml poly-d-lysine (Merck KGaA) at a density of 700 cells per mm^2^. Neurons were cultivated in Neurobasal Medium(Gibco, Thermo Scientific) containing 2% B-27 supplement (Gibco, Thermo Scientific), 100 IU/ml penicillin/streptomycin (Gibco, Thermo Scientific), 100 μg/ml Primocin (InvivoGen), and 0.5 mm glutamine (Life Technologies, Thermo Scientific) and maintained at 37 °C, 5.8% CO_2_, and 95% humidified atmosphere. Half of the nutrition medium was exchanged every 2 days and experiments were performed 7 days after neuron dissection.

##### Western Blotting Analysis

Protein expression was analyzed as previously described (7). Antibodies against MGL (polyclonal, in-house made rabbit anti-MGL serum (7), GFAP (ProSci, Flint Palace, CA), or GAPDH (Cell Signaling, Danvers, MA) and the respective horseradish peroxidase conjugated secondary antibodies were used.

##### Determination of in Vitro Monoglyceride Hydrolase (MGH) Activities

MGH activities of tissues and cells were determined as previously described (7). For MGL inhibition studies, samples were pretreated with either DMSO or 1 μm JZL184 (Cayman Chemical, Ann Arbor, MI) for 10 min at 37 °C.

##### Metabolic Phenotyping

For determination of food intake, locomotor activity, oxygen consumption, and respiratory quotient of mice, the Lab Master phenotyping platform (TSE Systems, Bad Homburg, Germany) was used. Therefore, 3-month-old mice were accustomed to Lab Master drinking bottles for at least 3 days in their home cages. Subsequently, mice were single housed and acclimatized to phenotyping cages for 3 days before measurement.

##### Behavioral Testing

The open field test was performed as described (21). Mice were individually placed in the center of the open field, and their behavior during a 5 min test period was tracked by a video camera and recorded with the VideoMot2 software (TSE Systems).

For the tail suspension test, mice were suspended by their tail with strapping tape (Leukotape classic; BSN Medical S.A.S., Le Mans, France) to a lever for 6 min, and their behavior was recorded and analyzed via VideoMot2 software (TSE Systems). A trained blinded observer analyzed the video recordings with the event monitoring module for 3 types of behavior: swinging, curling, and immobility (22, 23).

The social memory test was performed in a three-chambered apparatus (24, 25). Mouse behavior was tracked with the VideoMot2 software (TSE Systems) during a 5-min test period evaluating the time spent in the immediate vicinity (5 cm) of the grid enclosures and number of entries into each.

Acute thermal nociception was assessed with a Plantar Test apparatus (model 7370, Ugo Basile, Comerio, Italy) as described (26, 27). The mean paw withdrawal latencies for both hind paws were calculated from the average of 3 separate trials, taken at 2-min intervals.

Spatial memory was analyzed using the Barnes maze (28). All sessions were recorded and analyzed with the VideoMot2 software (TSE Systems).

##### Lipid Analysis

For 2-AG analysis brains were homogenized and extracted twice with CHCl_3_/MeOH/H_2_O, (2:1:0.6, *v*/*v*/*v*) containing 500 nm butylated hydroxytoluene (BHT), 1% glacial acetic acid, and 1-heptadecanoyl-rac-glycerol (C17:0 monoglyceride (MG), Avanti Lipids) as internal standard. The lipid-containing organic phase was dried, and MGs were isolated by solid phase extraction using silica gel columns. Fractions were obtained by consecutive elution with 99/1, and 90/10 CHCl_3_/MeOH (*v*/*v*). The latter fraction containing MGs was used for 2-AG quantification using an AQUITY-UPLC (Waters, Manchester, UK) equipped with a BEH-C18-column (2.1 × 150 mm, 1.7 μm; Waters) coupled to a SYNAPT^TM^ G1 qTOF HD mass spectrometer (Waters) equipped with an ESI sources (29). Quantifier ions were MH+ and MNa+ (*m*/*z* 379, 401) for 2-AG and MH+ and MNa+ (*m*/*z* 327, 367) for C17:0-MG, respectively. 2-AG of 0.5–100 pmol/μl together with C17:0-MG was used for the calibration curve. For prostaglandin E2 (PGE2) and AA analysis, mouse brains were transferred into ZR BashingBeadLysis Tubes (Zymo Research), spiked with internal standard mixture (3.5 ng of each standard) in 1 ml of ice cold MeOH, containing 0.002% butylated hydroxytoluene and immediately homogenized with a MP FastPrep24 (MP Biomedicals) instrument. Samples were centrifuged at 4 °C for 10 min at 10,000 × *g*. The supernatant was removed and the extraction was repeated. Supernatants were merged and an aliquot was injected for metabolite separation via an Agilent 1290 UHPLC system (Agilent Technologies) using a BEH C18 reverse-phase column (100 × 2.1 mm, 1.7 μm, Waters). A linear gradient of solvent A (0.1% acetic acid) and solvent B (acetonitrile/isopropanol/acetic acid 95:5:0.1, *v*/*v*/*v*) starting from 5% solvent B and linear increasing to 95% solvent B was performed over 12 min with a flow rate of 0.55 ml/min. PGE2 and AA levels were quantified by multi reaction monitoring of each metabolite using an Agilent G6460A QQQ mass spectrometer (Agilent Technologies) with MS parameters as previously described (30).

##### Immunohistochemistry (IHC) and Immunofluorescence (IF)

IHC and IF were performed as previously described (31). In brief, mice were deeply anesthetized and transcardially perfused with PBS and 4% paraformaldehyde (PFA). Brains were excised, postfixed in 4% PFA overnight, saturated in 30% sucrose solution, and sectioned with a cryotome at 30 μm. IHC staining of microglia was performed using anti-IBA1 antibody (Wako Chemicals, Neuss, Germany) and imaged using a Zeiss AXIO Scope A1 equipped with a Plan-APOCHROMAT 20×/0.8 objective. Images were acquired at room temperature with the AxioRel Vision 4.8 software and quantified in ImageJ. For double IF goat anti-GFAP antibody (Sigma-Aldrich) and rabbit anti-MGL antibody (kind gift from Ken Mackie, Indiana University Bloomington) and anti-goat (Alexa Fluor 488-conjugated) and anti-rabbit (Alexa Fluor 594-conjugated) secondary antibodies were used. Sections were imaged by confocal laser scanning microscopy using a Leica TCS SP5 II equipped with a HCX PL APO 63 × 1.3 NA objective. Images were obtained at room temperature with Leica LAS AF software.

##### Cytokine Determination

Tissues were excised, briefly washed in PBS, snap frozen in liquid N_2_ within 30 s, and stored at −80 °C. For cytokine measurements tissues were homogenized in 1 ml of PBS + protease inhibitor (cOmplete Protease Inhibitor Mixture Tablets, Roche) and centrifuged at 1,000 × *g* for 10 min at 4 °C. The supernatant was transferred into a new tube and centrifuged at 100,000 × *g* for 40 min at 4 °C. For determination of cytokine levels the Custom Mix 'N Match Multi-Analyte ELISArray (Qiagen, Hilden, Germany) was used according to the manufacturer's protocol. In brief, ELISA wells were incubated with antigen standards or 100 μg sample for 2 h at room temperature, washed, and incubated with respective Biotin labeled detection antibodies for 1 h at room temperature. After washing wells were incubated for 30 min with Avidin-HRP at room temperature. Subsequently, wells were incubated with development solution for 15 min at room temperature followed by addition of stop solution and measurement of absorbance at 450 nm. For quantification readings at 567 nm were subtracted.

##### Statistical Analysis

Values are represented as means ± S.D. Statistical significance was determined by Student's unpaired *t* test (two-tailed) for the comparison of genotypes. For analysis of multiple measurements, analysis of variance followed by Bonferroni *post hoc* test was used. Group differences were considered statistically significant for genotypes: *, *p* < 0.05; **, *p* < 0.01; and ***, *p* < 0.001; for treatments: #, *p* < 0.05; ##, *p* < 0.01; and ###, *p* < 0.001.

## Results

### 

#### 

##### Generation and Gross Characterization of Mice with Astrocyte-specific Deletion of MGL

**FIGURE 1.**
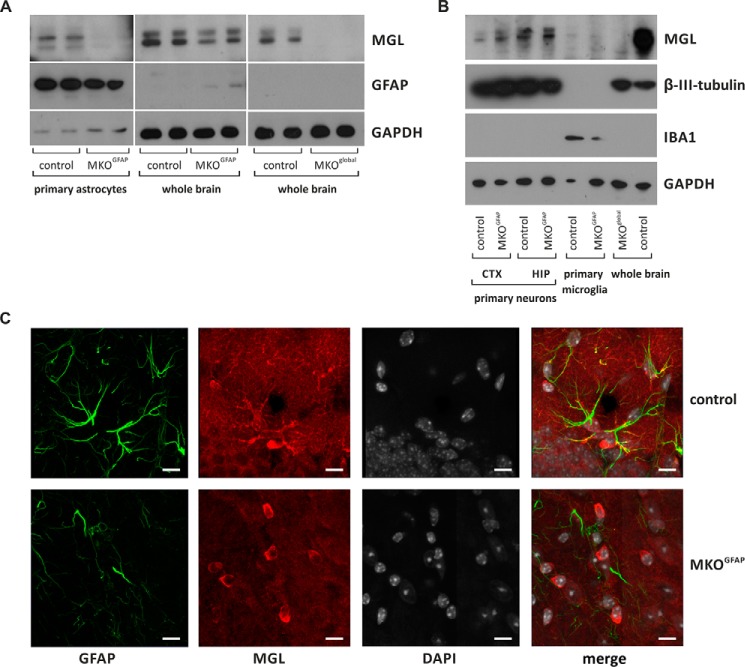
**MGL expression in MKO^GFAP^ mice.** *A*, Western blot analysis revealed absent MGL expression in lysates of primary cultivated astrocytes from MKO^GFAP^ mice. Minor reductions were observed in lysates of MKO^GFAP^ brains. MKO^global^ brains served as control and showed no MGL expression. As controls we used floxed littermates for MKO^GFAP^ mice and wild-type littermates for MKO^global^ mice. *B*, MGL is expressed in primary neurons from cortex (*CTX*) and hippocampus (*HIP*) of MKO^GFAP^ mice. Low expression was observed in primary microglia. Brain lysates of wild-type and MKO^global^ mice served as positive and negative controls, respectively. *C*, immunofluorescence images of MGL (*red*) and GFAP (*green*) of brain cortical sections obtained from floxed control and MKO^GFAP^ mice. Nuclei (*white*) were stained with DAPI. The scale bar represents 10 μm.

**FIGURE 2.**
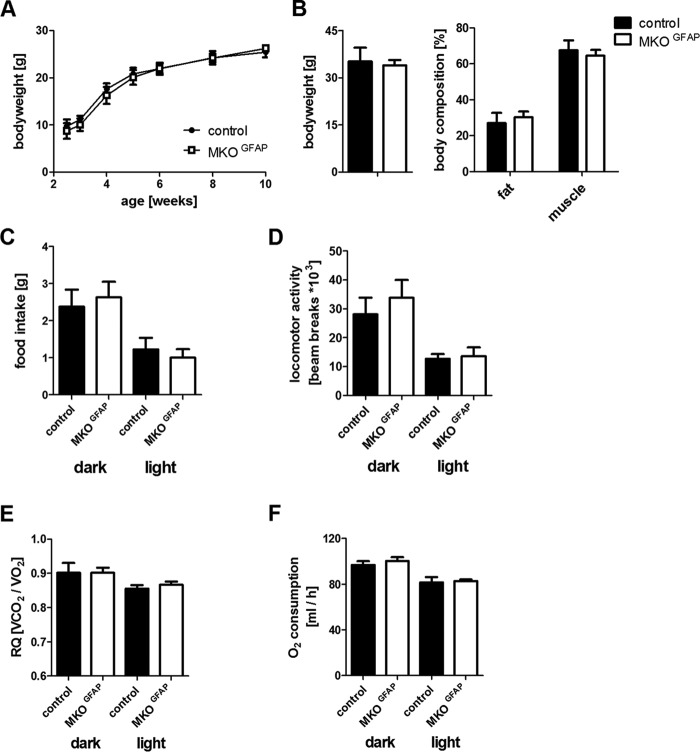
**Basic characterization of MKO^GFAP^ mice.** *A*, bodyweight during development. *B*, bodyweight and body composition of 3-month-old mice. Body composition was analyzed using a NMR Microspectrometer (the minispec, NMR Analyzer, Bruker, Ettlingen, Germany). *C*, food consumption; *D*, locomotor activity; *E*, respiratory quotient; and *F*, O_2_ consumption of floxed control and MKO^GFAP^ mice were calculated from a 96 h measurement period in a laboratory animal monitoring system (LabMaster, TSE Systems). Data are presented as means + S.D. (*n* = 6).

Astrocyte-specific MGL knock-out mice (MKO^GFAP^) were generated by crossing MGL-floxed animals to mice expressing Cre-recombinase under control of the hGFAP-promotor. Primary astrocytes from MKO^GFAP^ mice exhibited markedly reduced MGL protein expression, whereas a minor reduction in MGL expression was observed in whole brain lysates (Fig. 1*A*). Brain lysates of MKO^global^ mice showed no MGL expression confirming the specificity of the antibody (Fig. 1*A*). In contrast to astrocytes, MGL protein expression was preserved in primary neurons from cortex and hippocampus. In accordance with published data, primary microglia exhibited low MGL expression (32), which was barely detectable in cultures obtained from control and MKO^GFAP^ mice (Fig. 1*B*). To confirm astrocyte-specificity of MGL deletion, we performed double immunofluorescence analysis of MGL and the astrocyte marker GFAP. In cortical brain sections of control mice, we observed a co-localization of MGL and GFAP-positive cells (Fig. 1*C*). Although MGL expression was detected in sections of MKO^GFAP^ mice, we could not observe any co-localization of MGL and GFAP (Fig. 1*C*). Together, these observations implicate that MKO^GFAP^ mice lack MGL specifically in astrocytes. Gross phenotypic characterization revealed that MKO^GFAP^ mice exhibit no alterations in bodyweight, body composition, food intake, locomotor activity, oxygen consumption, and respiratory quotient (RQ) (Fig. 2).

##### Astrocyte-specific Deletion of MGL Reduces Total Brain Monoglyceride Hydrolase Activity and Increases 2-AG Levels

**FIGURE 3.**
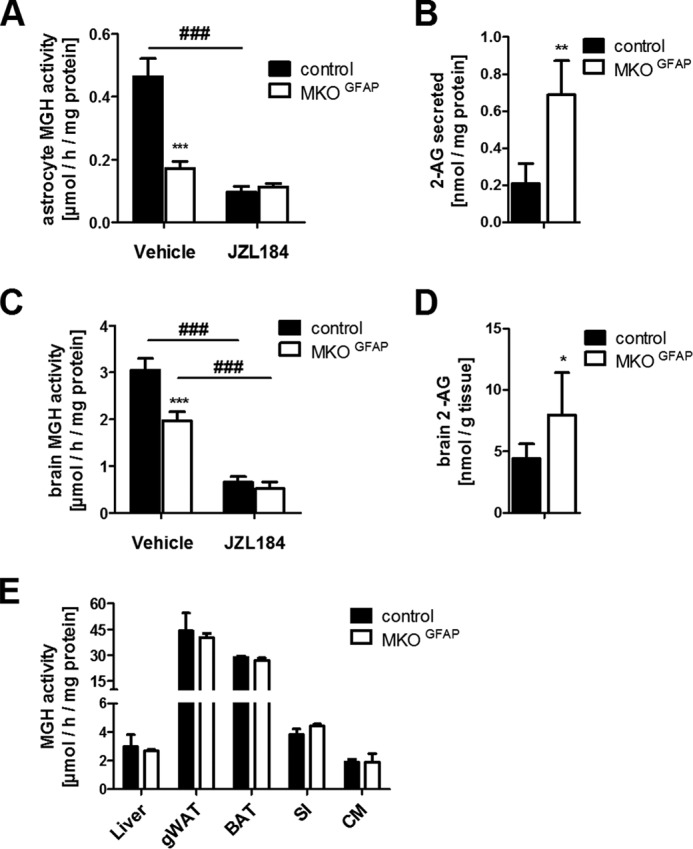
**MGH activity and 2-AG levels.** *In vitro* MGH activities of **(***A*) primary astrocytes or (*C*) whole brain lysates from floxed control and MKO^GFAP^ mice. Activities were determined in presence of vehicle (DMSO) or 1 μm JZL184. ***, *p* < 0.001; ###, *p* < 0.001 *versus* respective vehicle control (analysis of variance, Bonferroni post hoc test) (*n* = 3). *B*, 2-AG levels were measured from the medium of astrocyte cultures or (*D*) from total brain of floxed control and MKO^GFAP^ mice via UPLC-MS (*n* = 4–6). *E*, *in vitro* MGH activities of lysates from peripheral tissues of floxed control and MKO^GFAP^ mice (*n* = 3). Data are presented as means + S.D. *, *p* < 0.05; **, *p* < 0.01; ***, *p* < 0.001 (Student's *t* test).

Next, we determined the impact of MGL deletion on *in vitro* MGH activity in isolated primary astrocytes. In lysates of MKO^GFAP^ astrocytes, we found 65% reduction in MGH activity as compared with controls. Addition of the MGL inhibitor JZL184 (1 μm) reduced MGH activity of control and MKO^GFAP^ astrocytes to 20% of total activity observed in controls (Fig. 3*A*). The reduced MGH activity led to a 3-fold increase in 2-AG levels in the medium of MKO^GFAP^ astrocytes (Fig. 3*B*). To assess the contribution of astrocyte MGL to total brain MGH activity, we compared activities detected in brain lysates of control and MKO^GFAP^ mice. As shown in Fig. 3*C*, MKO^GFAP^ mice exhibited 35% reduction in total brain MGH activity. Addition of the MGL inhibitor JZL184 (1 μm) reduced MGH activity in MKO^GFAP^ and control lysates to 15% of total activity observed in controls (Fig. 3*C*). Reduced MGH activity led to a 1.8-fold increase in total brain 2-AG levels as compared with controls (Fig. 3*D*). In accordance with the tissue specificity of the hGFAP promotor (19), no differences in MGH activities were observed in lysates from liver, gonadal white adipose tissue, brown adipose tissue, small intestine, and cardiac muscle (Fig. 3*E*).

##### Global but Not Astrocyte-specific MGL Deletion Leads to CBR Desensitization

**FIGURE 4.**
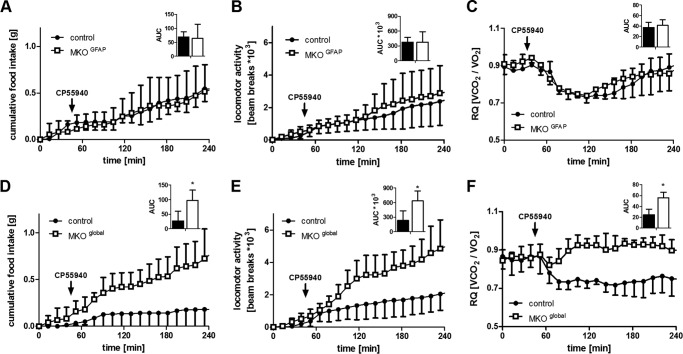
**Acute response of MKO^GFAP^ and MKO^global^ mice upon CBR agonist treatment.** Mice were housed in a laboratory animal monitoring system (LabMaster, TSE Systems). *A*, *D*, food intake; *B*, *E*, locomotor activity; and *C*, *F*, respiratory quotient (RQ) were monitored after intraperitoneal injection of 0.15 mg/kg CBR agonist CP55940 (dissolved in 0.9% saline containing 5% ethanol and 5% Cremophor®). *Arrows* indicate time point of injection. Mice were familiarized with cages for 3 days before measurement. As controls we used floxed littermates for MKO^GFAP^ mice and wild-type littermates for MKO^global^ mice. Data are presented as mean ± S.D. (*n* = 4–6). *, *p* < 0.05 (Student's *t* test).

As shown in previous studies, global genetic deletion or chronic pharmacological inhibition of MGL results in CBR desensitization (5, 6). We and others previously demonstrated that MKO^global^ mice are resistant against the hypometabolic effects of the CBR agonist CP55940 due to 2-AG dependent CBR desensitization (6, 7). Thus, we investigated whether moderately elevated 2-AG levels in the brain of MKO^GFAP^ mice affect CBR sensitivity. Administration of CP55940 (0.15 mg/kg, intraperitoneal) led to a similar reduction of food intake (Fig. 4*A*), locomotor activity (Fig. 4*B*), and RQ (Fig. 4*C*) in control and MKO^GFAP^ mice, indicating that CBR sensitivity in MKO^GFAP^ mice is not affected. As a control, we equally treated MKO^global^ mice, and found that these mice exhibit increased food intake (Fig. 4*D*), locomotor activity (Fig. 4*E*), and RQ (Fig. 4*F*) in comparison to wild-type controls. These observations confirm that global MGL deficiency leads to functional antagonism of the endocannabinoid system and indicate that the moderate increase in 2-AG levels in the brain of MKO^GFAP^ mice is not sufficient to cause CBR desensitization.

##### Astrocyte-specific MGL Deletion Does Not Cause Cannabimimetic Behavioral Effects

**FIGURE 5.**
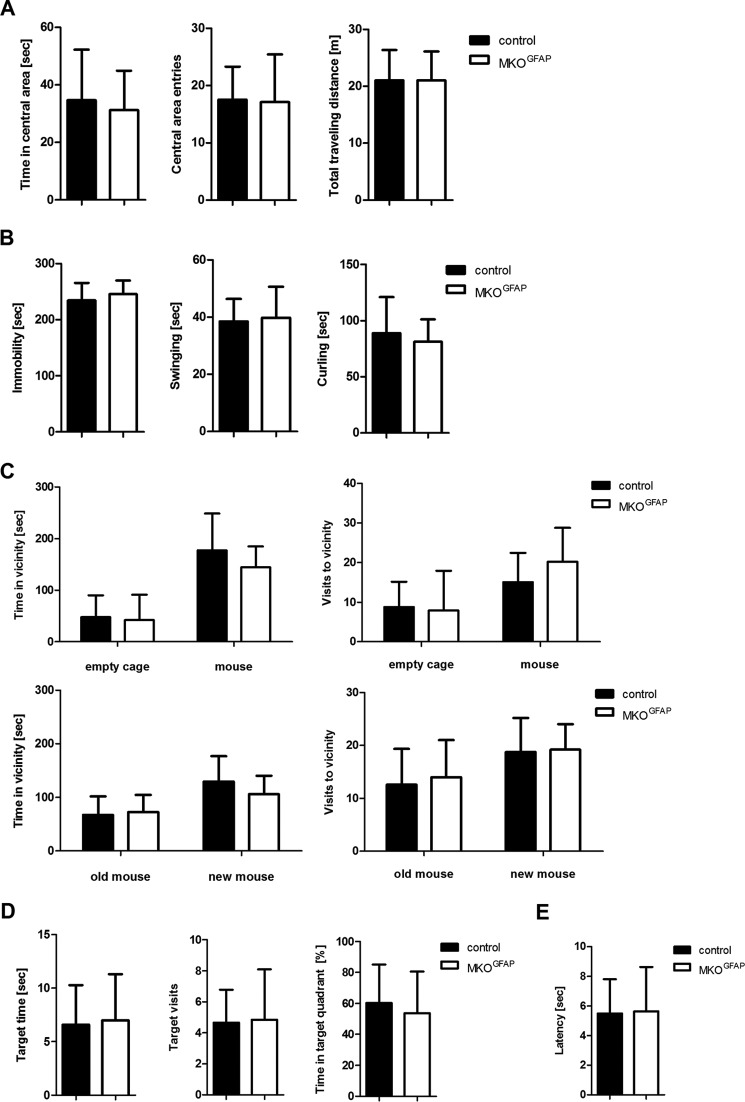
**Behavioral characterization of MKO^GFAP^ mice.** Floxed control and MKO^GFAP^ mice were tested for (*A*) anxiety-like behavior in the open field test and (*B*) depression-like behavior in the tail suspension test. *C*, social memory was analyzed via social interaction test and *D*, spatial memory via Barnes maze. *E*, thermal nociception was tested via plantar test. Data are presented as means + S.D. (*n* = 10–12).

To investigate whether increased 2-AG levels cause cannabimimetic effects, MKO^GFAP^ mice were subjected to several behavioral tests. Yet, we could not observe any differences in anxiety-like behavior in the open field test (Fig. 5*A*), depression-like behavior in the tail suspension test (Fig. 5*B*), social interaction (Fig. 5*C*), spatial memory in the Barns maze (Fig. 5*D*), or thermal nociception (Fig. 5*E*). These data implicate that deletion of MGL in astrocytes is not sufficient to cause major behavioral changes as observed in response to cannabinoid treatment (33).

##### Astrocyte-specific MGL Deletion Attenuates LPS Induced Neuroinflammation

**FIGURE 6.**
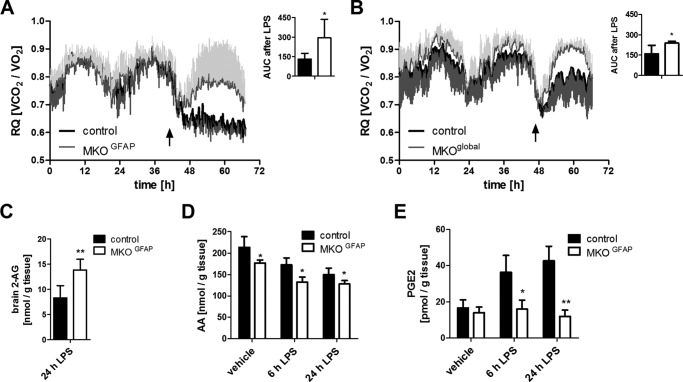
**Neuroinflammatory response of MKO^GFAP^ mice after peripheral LPS treatment.** *A*, MKO^GFAP^ and *B*, MKO^global^ mice were housed in a laboratory animal monitoring system (LabMaster, TSE Systems), and RQ was monitored after intraperitoneal injection of 5 mg/kg LPS. The *arrow* indicates the time point of injection. Mice were familiarized with cages for 3 days before treatment. *C*, brain 2-AG; *D*, AA; and *E*, prostaglandin E2 levels were analyzed 6 h or 24 h after intraperitoneal administration of 5 mg/kg LPS. As controls we used floxed littermates for MKO^GFAP^ mice and wild-type littermates for MKO^global^ mice. Data are presented as means ± S.D. (*n* = 4–6). *, *p* < 0.05; **, *p* < 0.01; (Student's *t* test).

**FIGURE 7.**
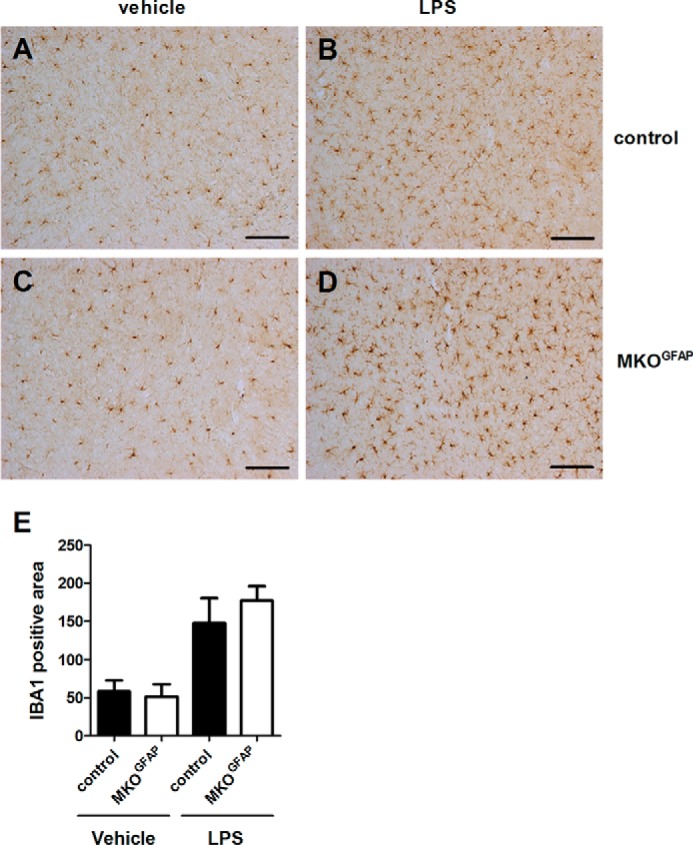
**Microglia activation in MKO^GFAP^ mice upon LPS treatment.** Mice were treated with vehicle or 0.5 mg/kg LPS in saline on 4 consecutive days to induce microglia activation. Representative images of microglia morphology in cortical brain sections of floxed control (*A*, *B*) and MKO^GFAP^ mice (*C*, *D*). Mice were either treated with (*A*, *C*) vehicle or (*B*, *D*) LPS. IHC was performed using the microglial marker IBA1. The scale bar represents 100 μm. *E*, relative microglia density was determined by quantification of IBA1 positive areas in 5–7 cortical brain sections of each mouse (3 mice per genotype and treatment) with ImageJ software. Data are represented as means ± S.D.

Published data suggest that global deletion of MGL in mice has beneficial effects on neuroinflammation by reducing the amount of AA available for prostaglandin synthesis (12). Likewise, 2-AG has been shown to have neuroprotective properties *per se* (9). Since astrocytes play a major role in neuroinflammation, we hypothesized that astrocyte-specific deletion of MGL has similar effects on neuroinflammation as observed in MKO^global^ mice. To induce systemic inflammation, mice were treated with LPS (5 mg/kg, intraperitoneal). Subsequently, we monitored LPS induced sickness behavior using the RQ as sickness index, which is reduced in response to LPS treatment (34). As shown in Fig. 6*A*, control mice showed a pronounced reduction in RQ upon LPS administration and this effect was attenuated in MKO^GFAP^ mice. We confirmed the beneficial effect of global MGL deficiency in LPS induced sickness in MKO^global^ mice (Fig. 6*B*). Next, we investigated brain 2-AG, AA, PGE2, and cytokine levels. LPS treated MKO^GFAP^ mice exhibited a ∼2-fold increase in brain 2-AG (Fig. 6*C*) similar as observed under basal conditions (Fig. 3*D*). Brain AA levels in vehicle treated MKO^GFAP^ mice were reduced by ∼20% in comparison to wild-type controls and further decreased slightly in response to LPS treatment in both control and MKO^GFAP^ mice (Fig. 6*D*). Notably, LPS substantially increased brain PGE2 levels in control but not in MKO^GFAP^ mice (Fig. 6*E*). This observation implicates that MGL activity in astrocytes strongly determines the availability of AA for prostaglandin synthesis upon inflammatory stimuli. LPS treatment leads to activation of brain microglia, which are important in the inflammatory response of the CNS (35). To test the role of astrocyte MGL in microglia activation, we treated mice with 0.5 mg/kg LPS on 4 consecutive days to induce microglia activation as previously described for MKO^global^ mice (12). Microglia morphology and density was determined via IHC of cortical brain sections using the microglia marker IBA1. As shown in representative images, microglia morphology and density was similar in vehicle-treated control (Fig. 7*A*) and MKO^GFAP^ (Fig. 7*C*) mice. Microglia were similarly activated in both genotypes after LPS treatment (Fig. 7, *B* and *D*). Accordingly, quantification of IBA1 positive area revealed unchanged microglia density in both genotypes (Fig. 7*E*).

**FIGURE 8.**
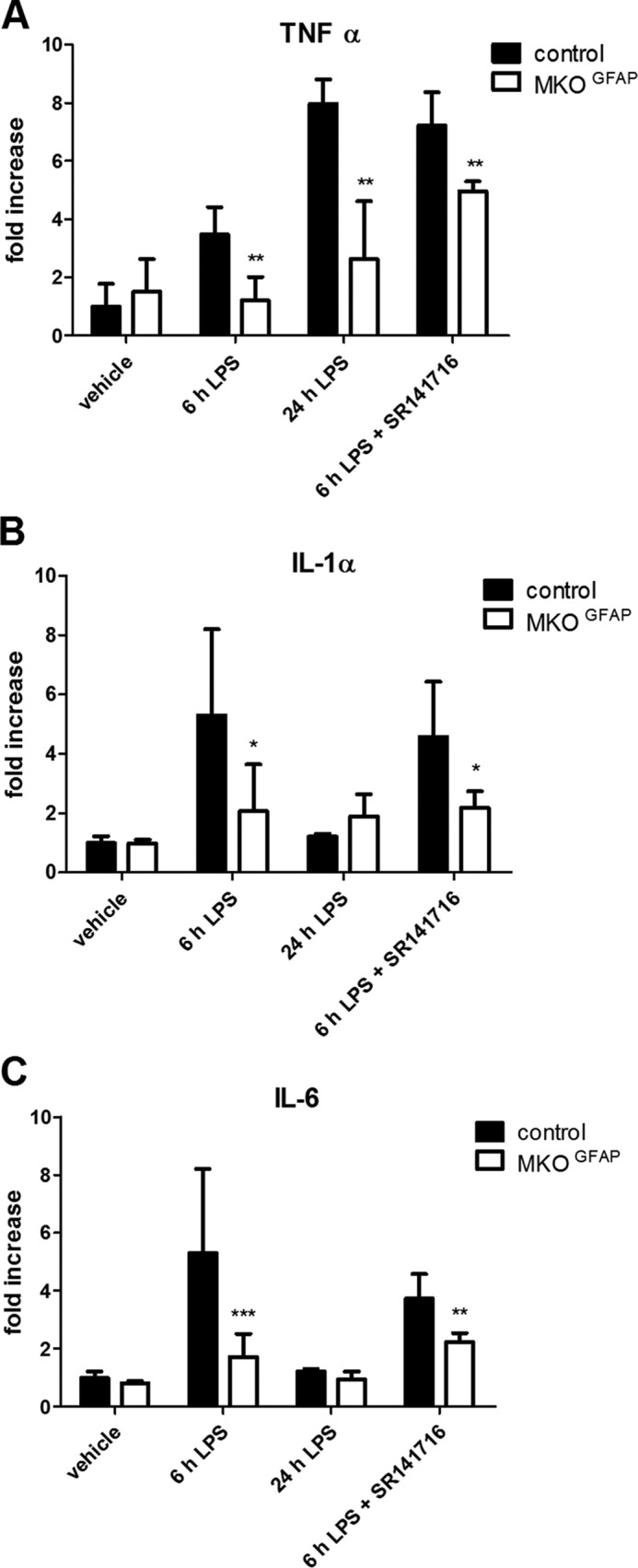
**Brain cytokines of MKO^GFAP^ mice upon LPS treatment.** *A*, TNFα; *B*, IL-1α; and *C*, IL-6 levels were determined in cytosolic brain fractions of floxed control and MKO^GFAP^ mice using the Custom Mix-N-Match ELISArray (Qiagen) 6 or 24 h after intraperitoneal injection of 5 mg/kg LPS. Mice were treated with vehicle or SR141716 (1 mg/kg, intraperitoneal) 1 h before LPS administration. Data are presented as means + S.D. (*n* = 4–6). *, *p* < 0.05; **, *p* < 0.01; ***, *p* < 0.001 (Student's *t* test).

Next, we assessed whether reduced PGE2 is associated with altered cytokine production. LPS treatment induced a time-dependent increase in cytokines TNFα (Fig. 8*A*), IL-1α (Fig. 8*B*), and IL-6 (Fig. 8*C*) in the brain of control mice and this effect was blunted in MKO^GFAP^ mice. Finally, we investigated whether the attenuated neuroinflammatory response in MKO^GFAP^ mice is dependent on CBR1 activation. We therefore treated mice with the inverse CBR1 agonist SR141716 (Rimonabant; 1 mg/kg, intraperitoneal) (36) 1 h before LPS administration. As shown in Fig. 8, *A–C*, TNFα, IL-1α, and IL-6 levels remained reduced in MKO^GFAP^ mice treated with SR141716 indicating that the reduction in neuroinflammation is rather independent of CBR1 activation. Taken together, our observations demonstrate that MGL in astrocytes is crucial for providing AA for prostaglandin synthesis upon LPS-induced neuroinflammation.

## Discussion

MGL is required for efficient degradation of 2-AG in brain and thus inversely regulates 2-AG levels and AA availability (1). This distinct metabolic function makes MGL an interesting target for the treatment of neuroinflammation, since 2-AG exhibits anti-inflammatory properties and AA is a precursor of pro-inflammatory prostaglandins (37). Accordingly, it has been shown that pharmacological or global genetic inactivation of MGL has beneficial effects in animal models of neuroinflammatory and neurodegenerative diseases (12, 38).

In the present study, we generated mice with genetic deletion of MGL specifically in astrocytes and investigated the impact on brain 2-AG and AA metabolism, behavior, and inflammation. MKO^GFAP^ mice exhibit only minor reduction in total brain MGH activities and a moderate increase of brain 2-AG levels as compared with MKO^global^ mice showing more than 10-fold increased brain 2-AG levels (5–7). The strongly elevated 2-AG levels in MKO^global^ mice cause tolerance to CBR agonists, which is also observed upon chronic pharmacological MGL inhibition or prolonged exposure to CBR agonists (6). The observed tolerance is caused by CBR desensitization in the brain and also in peripheral tissues such as the intestine (39). We previously demonstrated that measurement of physiological parameters in metabolic cages upon CBR agonist treatment can be used for determination of CBR desensitization *in vivo* (7). In contrast to MKO^global^ mice, MKO^GFAP^ mice respond to CBR agonist treatment similar as wild-type controls suggesting that the moderate increase in 2-AG in MKO^GFAP^ mice is not sufficient to cause CBR desensitization. Thus, 2-AG accumulation could affect animal behavior, since the endocannabinoid system plays a key role in learning, memory, anxiety, depression, and pain (33, 40–42). Additionally, CBR dependent signaling between astrocytes and neurons is involved in the regulation of synaptic plasticity and memory formation (43, 44). Therefore, we performed a variety of standard tests for social and spatial memory, anxiety and depression like behavior, and thermal nociception. However, we could not find any differences between control and MKO^GFAP^ mice. We conclude that the moderate increase of 2-AG in MKO^GFAP^ mice is not sufficient to elicit cannabimimetic behavioral effects.

Astrocytes are considered as major source of AA in brain (15, 45) and published data implicate that rather MGL than cytosolic phospholipase A2 controls the availability of AA for prostaglandin synthesis in the brain (12). Accordingly, we hypothesized that MGL in astrocytes may be crucial in providing AA for prostaglandin production and that MGL deletion has beneficial effects on neuroinflammation. Our observations demonstrate that deletion of MGL in astrocytes is sufficient to improve sickness behavior of mice in response to LPS treatment similar as observed for MKO^global^ mice. To reveal the underlying mechanisms, we measured 2-AG and AA levels. We found that 2-AG is moderately increased in the brain of MKO^GFAP^ mice under basal and inflammatory conditions. In accordance with previous studies, peripheral LPS administration altered brain 2-AG levels neither in wild-type (12) nor in MGL^GFAP^ mice. Free AA levels were moderately decreased in MKO^GFAP^ mice in comparison to wild-type controls and further decreased slightly in both genotypes upon LPS treatment. Importantly, MGL^GFAP^ mice completely lacked the LPS-induced increase in PGE2 synthesis and exhibited reduced levels of pro-inflammatory cytokines. Dysregulated PGE2 synthesis is associated with the onset and progression of neuroinflammatory and neurodegenerative diseases (46). Our data indicate that MGL in astrocytes is crucial for providing AA as substrate for PGE2 synthesis. These observations support the recently discovered role of MGL as interesting pharmacological target for treatment of neuroinflammation, providing a novel strategy for reduction of prostaglandin synthesis independent of COX inhibition (12). Several studies demonstrated the beneficial effects of COX1 inhibition in inflammatory processes of the CNS (47–49). Both COX1 and MGL inhibitors exhibit anti-inflammatory and analgesic properties (37, 50). Use of MGL inhibitors, however, can avoid gastrointestinal side effect of non steroidal anti-inflammatory drugs (NSAIDs) and even improve GI bleeding induced by NSAIDs (51).

Both astrocytes and microglia are thought to contribute to the onset and progression of neuroinflammation by producing pro-inflammatory mediators, whereby prostaglandins predominantly derive from activated microglia (52). Notably, microglia activation is unchanged in control and MKO^GFAP^ mice after LPS treatment. Very recent data support our observations by demonstrating that the endocannabinoid dependent metabolic interplay between neurons and astrocytes is involved in the regulation of inflammatory processes of the CNS. Using an inducible knock-out system, Viader *et al.* demonstrate that rather MGL in astrocytes than in microglia or neurons affects neuroinflammation in response to single and repeated LPS treatment (53).

To investigate whether increased 2-AG levels in MKO^GFAP^ mice contribute to the anti-inflammatory effects, we treated mice with the inverse CBR1 agonist SR141716. Interestingly, pro-inflammatory cytokine levels remained reduced in LPS-treated MKO^GFAP^ mice, after SR141716 treatment. This suggests that the reduced mobilization of AA for prostaglandin synthesis rather than the accumulation of 2-AG is responsible for the beneficial effects on neuroinflammation. Accordingly, studies in CBR1/CBR2 double knock-out mice implicate that neuroprotective effects of MGL inhibition are independent of CBR (12). Nevertheless, another study demonstrates that CBR1 mediates the hypothermic effects of LPS in rats (54). Thus, the activation of CBRs and the reduced availability of AA might regulate different aspects of LPS-induced inflammation.

In summary, we demonstrate that MGL in astrocytes affects brain 2-AG hydrolysis, AA availability, and prostaglandin synthesis. Astrocyte-specific deletion of MGL is not sufficient to cause cannabimimetic behavioral effects or CBR desensitization, but reproduces the neuroprotective effects of global MGL deficiency.

## Author Contributions

G. F. G., Y. G., U. T., and F. P. W. R. performed experiments and analyzed the results. T. O. E. and B. W. performed lipid analysis. A. F. and P. H. conducted and analyzed behavioral tests. M. S., A. L., E. Z., M. H. T., C. Y., and R. Z. supervised the experiments and analyzed the results. G. F. G. and R. Z. designed the study and wrote the manuscript. All authors reviewed and approved the final version of the manuscript.

## Footnotes

2 The abbreviations used are:

MGL: monoglyceride lipase
2-AG: 2-arachidonoylglycerol
AA: arachidonic acid
CBR: cannabinoid receptor
GFAP: glial fibrillary acidic protein
MG: monoglyceride
UPLC: ultra high performance liquid chromatography
IHC: immunohistochemistry
IF: immunofluorescence
DMSO: dimethyl sulfoxide
RQ: respiratory quotient
PGE2: prostaglandin E2.

## References

[1] Dinh T. P., Freund T. F., and Piomelli D. (2002) A role for monoglyceride lipase in 2-arachidonoylglycerol inactivation. Chem. Phys. Lipids. 121, 149–158 12505697 10.1016/s0009-3084(02)00150-0

[2] Stella N. (2004) Cannabinoid signaling in glial cells. Glia 48, 267–277 15390110 10.1002/glia.20084

[3] Blankman J. L., Simon G. M., and Cravatt B. F. (2009) A comprehensive profile of brain enzymes that hydrolyze the endocannabinoid 2-arachidonoylglycerol. Chem. Phys. Lipids 14, 1347–1356 10.1016/j.chembiol.2007.11.006 PMC2692834 18096503

[4] Matsuda L. A., Lolait S. J., Brownstein M. J., Young A. C., and Bonner T. I. (1990) Structure of a cannabinoid receptor and functional expression of the cloned cDNA. Nature 346, 183–187 2165569 10.1038/346561a0

[5] Chanda P. K., Gao Y., Mark L., Btesh J., Strassle B. W., Lu P., Piesla M. J., Zhang M., Bingham B., Uveges A., Kowal D., Garbe D., Kouranova E. V., Ring R. H., Bates B., Pangalos M. N., Kennedy J. D., Whiteside G. T., and Samad T. A. (2010) Monoacylglycerol lipase activity is a critical modulator of the tone and integrity of the endocannabinoid system. Mol. Pharmacol. 78, 996–1003 20855465 10.1124/mol.110.068304

[6] Schlosburg J. E., Blankman J. L., Long J. Z., Nomura D. K., Pan B., Kinsey S. G., Nguyen P. T., Ramesh D., Booker L., James J., Thomas E. A., Selley D. E., Sim-Selley L. J., Liu Q., Lichtman H., and Cravatt B. F. (2010) Chronic monoacylglycerol lipase blockade causes functional antagonism of the endocannabinoid system. Nat. Neurosci. 13, 1113–1119 20729846 10.1038/nn.2616 PMC2928870

[7] Taschler U., Radner F. P. W., Heier C., Schreiber R., Schweiger M., Schoiswohl G., Preiss-Landl K., Jaeger D., Reiter B., Koefeler H. C., Wojciechowski J., Theussl C., Penninger J. M., Lass A., Haemmerle G., Zechner R., and Zimmermann R. (2011) Monoglyceride lipase deficiency in mice impairs lipolysis and attenuates diet-induced insulin resistance. J. Biol. Chem. 286, 17467–17477 21454566 10.1074/jbc.M110.215434 PMC3093820

[8] Marsicano G., Goodenough S., Monory K., Hermann H., Eder M., Cannich A., Azad S. C., Cascio M. G., Gutiérrez S. O., van der Stelt M., López-Rodriguez M. L., Casanova E., Schütz G., Zieglgänsberger W., Di Marzo V., Behl C., and Lutz B. (2003) CB1 cannabinoid receptors and on-demand defense against excitotoxicity. Science 302, 84–88 14526074 10.1126/science.1088208

[9] Panikashvili D., Simeonidou C., Ben-Shabat S., Hanus L., Breuer A., Mechoulam R., and Shohami E. (2001) An endogenous cannabinoid (2-AG) is neuroprotective after brain injury. Nature 413, 527–531 11586361 10.1038/35097089

[10] Walter L., Franklin A., Witting A., Wade C., Xie Y., Kunos G., Mackie K., and Stella N. (2003) Nonpsychotropic cannabinoid receptors regulate microglial cell migration. J. Neurosci. 23, 1398–1405 12598628 10.1523/JNEUROSCI.23-04-01398.2003 PMC6742252

[11] Buczynski M. W., Dumlao D. S., and Dennis E. A. (2009) Thematic Review Series: Proteomics. An integrated omics analysis of eicosanoid biology. J. Lipid Res. 50, 1015–1038 19244215 10.1194/jlr.R900004-JLR200 PMC2681385

[12] Nomura D. K., Morrison B. E., Blankman J. L., Long J. Z., Kinsey S. G., Marcondes M. C. G., Ward A. M., Hahn Y. K., Lichtman A. H., Conti B., and Cravatt B. F. (2011) Endocannabinoid hydrolysis generates brain prostaglandins that promote neuroinflammation. Science 334, 809–813 22021672 10.1126/science.1209200 PMC3249428

[13] Walter L., Dinh T., and Stella N. (2004) ATP induces a rapid and pronounced increase in 2-arachidonoylglycerol production by astrocytes, a response limited by monoacylglycerol lipase. J. Neurosci. 24, 8068–8074 15371507 10.1523/JNEUROSCI.2419-04.2004 PMC6729797

[14] Araque A., Carmignoto G., Haydon P. G., Oliet S. H. R., Robitaille R., and Volterra A. (2014) Gliotransmitters travel in time and space. Neuron 81, 728–739 24559669 10.1016/j.neuron.2014.02.007 PMC4107238

[15] Moore S. A. (2000) Polyunsaturated fatty acid synthesis and release by brain-derived cells in vitro. J. Mol. Neurosci. 16, 195–200 11478374 10.1385/JMN:16:2-3:195

[16] Sofroniew M. V (2014) Multiple roles for astrocytes as effectors of cytokines and inflammatory mediators. Neuroscientist 20, 160–172 24106265 10.1177/1073858413504466

[17] Uchigashima M., Yamazaki M., Yamasaki M., Tanimura A., Sakimura K., Kano M., and Watanabe M. (2011) Molecular and morphological configuration for 2-arachidonoylglycerol-mediated retrograde signaling at mossy cell-granule cell synapses in the dentate gyrus. J. Neurosci. 31, 7700–7714 21613483 10.1523/JNEUROSCI.5665-10.2011 PMC6633146

[18] Harno E., Cottrell E. C., and White A. (2013) Metabolic pitfalls of CNS Cre-based technology. Cell Metab. 18, 21–28 23823475 10.1016/j.cmet.2013.05.019

[19] Zhuo L., Theis M., Alvarez-Maya I., Brenner M., Willecke K., and Messing A. (2001) hGFAP-cre transgenic mice for manipulation of glial and neuronal function *in vivo*. Genesis. 31, 85–94 11668683 10.1002/gene.10008

[20] Bajenaru M. L., Zhu Y., Hedrick N. M., Donahoe J., Parada L. F., and Gutmann D. H. (2002) Astrocyte-specific inactivation of the neurofibromatosis 1 gene (*NF1*) is insufficient for astrocytoma formation. Mol. Cell Biol. 22, 5100–5113 12077339 10.1128/MCB.22.14.5100-5113.2002 PMC139771

[21] Farzi A., Reichmann F., Meinitzer A., Mayerhofer R., Jain P., Hassan A. M., Fröhlich E. E., Wagner K., Painsipp E., Rinner B., and Holzer P. (2015) Synergistic effects of NOD1 or NOD2 and TLR4 activation on mouse sickness behavior in relation to immune and brain activity markers. Brain. Behav. Immun. 44, 106–120 25218901 10.1016/j.bbi.2014.08.011 PMC4295938

[22] Berrocoso E., Ikeda K., Sora I., Uhl G. R., Sánchez-Blázquez P., and Mico J. A. (2013) Active behaviours produced by antidepressants and opioids in the mouse tail suspension test. Int. J. Neuropsychopharmacol. 16, 151–162 22217458 10.1017/S1461145711001842

[23] Cryan J. F., Mombereau C., and Vassout A. (2005) The tail suspension test as a model for assessing antidepressant activity: review of pharmacological and genetic studies in mice. Neurosci. Biobehav. Rev. 29, 571–625 15890404 10.1016/j.neubiorev.2005.03.009

[24] Nadler J. J., Moy S. S., Dold G., Trang D., Simmons N., Perez A., Young N. B., Barbaro R. P., Piven J., Magnuson T. R., and Crawley J. N. (2004) Automated apparatus for quantitation of social approach behaviors in mice. Genes. Brain. Behav. 3, 303–314 15344923 10.1111/j.1601-183X.2004.00071.x

[25] Brunner S. M., Farzi A., Locker F., Holub B. S., Drexel M., Reichmann F., Lang A. A., Mayr J. A., Vilches J. J., Navarro X., Lang R., Sperk G., Holzer P., and Kofler B. (2014) GAL3 receptor KO mice exhibit an anxiety-like phenotype. Proc. Natl. Acad. Sci. U.S.A. 111, 7138–7143 24782539 10.1073/pnas.1318066111 PMC4024886

[26] Montagne-Clavel J., and Oliveras J. L. (1996) The “plantar test” apparatus (Ugo Basile Biological Apparatus), a controlled infrared noxious radiant heat stimulus for precise withdrawal latency measurement in the rat, as a tool for humans? Somatosens. Mot. Res. 13, 215–223 9110424 10.3109/08990229609052577

[27] Painsipp E., Wultsch T., Edelsbrunner M. E., Tasan R. O., Singewald N., Herzog H., and Holzer P. (2008) Reduced anxiety-like and depression-related behavior in neuropeptide Y Y4 receptor knockout mice. Genes. Brain. Behav. 7, 532–542 18221379 10.1111/j.1601-183X.2008.00389.x PMC4359911

[28] Attar A., Liu T., Chan W.-T. C., Hayes J., Nejad M., Lei K., and Bitan G. (2013) A shortened Barnes maze protocol reveals memory deficits at 4-months of age in the triple-transgenic mouse model of Alzheimer's disease. PLoS ONE. 8, e80355 24236177 10.1371/journal.pone.0080355 PMC3827415

[29] Knittelfelder O. L., Weberhofer B. P., Eichmann T. O., Kohlwein S. D., and Rechberger G. N. (2014) A versatile ultra-high performance LC-MS method for lipid profiling. J. Chromatogr. B. Analyt. Technol. Biomed. Life Sci. 951–952, 119–128 10.1016/j.jchromb.2014.01.011 PMC3946075 24548922

[30] Strassburg K., Huijbrechts A. M. L., Kortekaas K. A., Lindeman J. H., Pedersen T. L., Dane A., Berger R., Brenkman A., Hankemeier T., van Duynhoven J., Kalkhoven E., Newman J. W., and Vreeken R. J. (2012) Quantitative profiling of oxylipins through comprehensive LC-MS/MS analysis: application in cardiac surgery. Anal. Bioanal. Chem. 404, 1413–1426 22814969 10.1007/s00216-012-6226-x PMC3426673

[31] Yi C.-X., Tschöp M. H., Woods S. C., and Hofmann S. M. (2012) High-fat-diet exposure induces IgG accumulation in hypothalamic microglia. Dis. Model. Mech. 5, 686–690 22381575 10.1242/dmm.009464 PMC3424466

[32] Kouchi Z. (2015) Monoacylglycerol lipase promotes Fcγ receptor-mediated phagocytosis in microglia but does not regulate LPS-induced upregulation of inflammatory cytokines. Biochem. Biophys. Res. Commun. 10.1016/j.bbrc.2015.07.019 26166819

[33] Moreira F. A., and Lutz B. (2008) The endocannabinoid system: emotion, learning and addiction. Addict. Biol. 13, 196–212 18422832 10.1111/j.1369-1600.2008.00104.x

[34] Hollis J. H., Lemus M., Evetts M. J., and Oldfield B. J. (2010) Central interleukin-10 attenuates lipopolysaccharide-induced changes in food intake, energy expenditure and hypothalamic Fos expression. Neuropharmacology 58, 730–738 20045008 10.1016/j.neuropharm.2009.12.016

[35] Hoogland I. C. M., Houbolt C., van Westerloo D. J., van Gool W. A., and van de Beek D. (2015) Systemic inflammation and microglial activation: systematic review of animal experiments. J. Neuroinflammation. 12, 114 26048578 10.1186/s12974-015-0332-6 PMC4470063

[36] Rinaldi-Carmona M., Barth F., Héaulme M., Shire D., Calandra B., Congy C., Martinez S., Maruani J., Néliat G., and Caput D. (1994) SR141716A, a potent and selective antagonist of the brain cannabinoid receptor. FEBS Lett. 350, 240–244 8070571 10.1016/0014-5793(94)00773-x

[37] Mulvihill M. M., and Nomura D. K. (2013) Therapeutic potential of monoacylglycerol lipase inhibitors. Life Sci. 92, 492–497 23142242 10.1016/j.lfs.2012.10.025 PMC3594462

[38] Piro J. R., Benjamin D. I., Duerr J. M., Pi Y., Gonzales C., Wood K. M., Schwartz J. W., Nomura D. K., and Samad T. A. (2012) A dysregulated endocannabinoid-eicosanoid network supports pathogenesis in a mouse model of Alzheimer's disease. Cell Rep. 1, 617–623 22813736 10.1016/j.celrep.2012.05.001 PMC3715876

[39] Taschler U., Eichmann T. O., Radner F. P. W., Grabner G. F., Wolinski H., Storr M., Lass A., Schicho R., and Zimmermann R. (2015) Monoglyceride lipase deficiency causes desensitization of intestinal cannabinoid receptor type 1 and increased colonic μ-opioid receptor sensitivity. Br. J. Pharmacol. 172, 4419–29 26075589 10.1111/bph.13224 PMC4556478

[40] Long J. Z., Weiwei L., Lamont B., Burston J., Kinsey S. G., Schlosburg J. E., Pavón F. J., Serrano A. M., Selley D. E., and Loren H. (2009) Selective blockade of 2-arachidonoylglycerol hydrolysis produces cannabinoid behavioral effects. Nat. Chem. Biol. 5, 37–44 19029917 10.1038/nchembio.129 PMC2605181

[41] Ruehle S., Rey A. A., Remmers F., and Lutz B. (2012) The endocannabinoid system in anxiety, fear memory and habituation. J. Psychopharmacol. 26, 23–39 21768162 10.1177/0269881111408958 PMC3267552

[42] Riebe C. J., and Wotjak C. T. (2011) Endocannabinoids and stress. Stress 14, 384–397 21663537 10.3109/10253890.2011.586753

[43] Min R., and Nevian T. (2012) Astrocyte signaling controls spike timing-dependent depression at neocortical synapses. Nat. Neurosci. 15, 746–753 22446881 10.1038/nn.3075

[44] Han J., Kesner P., Metna-Laurent M., Duan T., Xu L., Georges F., Koehl M., Abrous D. N., Mendizabal-Zubiaga J., Grandes P., Liu Q., Bai G., Wang W., Xiong L., Ren W., Marsicano G., and Zhang X. (2012) Acute cannabinoids impair working memory through astroglial CB1 receptor modulation of hippocampal LTD. Cell 148, 1039–1050 22385967 10.1016/j.cell.2012.01.037

[45] Moore S. A., Yoder E., Murphy S., Dutton G. R., and Spector A. A. (1991) Astrocytes, not neurons, produce docosahexaenoic acid (22:6w-3) and arachidonic acid(20:4w-6). J. Neurochem. 56, 518–524 1824862 10.1111/j.1471-4159.1991.tb08180.x

[46] Rosenberger T. A., Villacreses N. E., Hovda J. T., Bosetti F., Weerasinghe G., Wine R. N., Harry G. J., and Rapoport S. I. (2004) Rat brain arachidonic acid metabolism is increased by a 6-day intracerebral ventricular infusion of bacterial lipopolysaccharide. J. Neurochem. 88, 1168–1178 15009672 10.1046/j.1471-4159.2003.02246.x

[47] Choi S.-H., Langenbach R., and Bosetti F. (2008) Genetic deletion or pharmacological inhibition of cyclooxygenase-1 attenuate lipopolysaccharide-induced inflammatory response and brain injury. FASEB J. 22, 1491–1501 18162486 10.1096/fj.07-9411com PMC2386977

[48] Choi S.-H., and Bosetti F. (2009) Cyclooxygenase-1 null mice show reduced neuroinflammation in response to β-amyloid. Aging 1, 234–244 20157512 10.18632/aging.100021 PMC2806008

[49] Matousek S. B., Hein A. M., Shaftel S. S., Olschowka J. A., Kyrkanides S., and O'Banion M. K. (2010) Cyclooxygenase-1 mediates prostaglandin E_2_ elevation and contextual memory impairment in a model of sustained hippocampal interleukin-1β expression. J. Neurochem. 114, 247–258 20412387 10.1111/j.1471-4159.2010.06759.x PMC2897946

[50] Crowe M. S., Leishman E., Banks M. L., Gujjar R., Mahadevan A., Bradshaw H. B., and Kinsey S. G. (2015) Combined inhibition of monoacylglycerol lipase and cyclooxygenases synergistically reduces neuropathic pain in mice. Br. J. Pharmacol. 172, 1700–1712 25393148 10.1111/bph.13012 PMC4376450

[51] Kinsey S. G., Nomura D. K., O'Neal S. T., Long J. Z., Mahadevan A., Cravatt B. F., Grider J. R., and Lichtman A. H. (2011) Inhibition of monoacylglycerol lipase attenuates nonsteroidal anti-inflammatory drug-induced gastric hemorrhages in mice. J. Pharmacol. Exp. Ther. 338, 795–802 21659471 10.1124/jpet.110.175778 PMC3164340

[52] Tzeng S.-F., Hsiao H.-Y., and Mak O.-T. (2005) Prostaglandins and cyclooxygenases in glial cells during brain inflammation. Curr. Drug Targets. Inflamm. Allergy 4, 335–340 16101543 10.2174/1568010054022051

[53] Viader A., Blankman J. L., Zhong P., Liu X., Schlosburg J. E., Joslyn C. M., Liu Q.-S., Tomarchio A. J., Lichtman A. H., Selley D. E., Sim-Selley L. J., and Cravatt B. F. (2015) Metabolic interplay between astrocytes and neurons regulates endocannabinoid action. Cell Rep. 12, 798–808 26212325 10.1016/j.celrep.2015.06.075 PMC4526356

[54] Steiner A. A., Molchanova A. Y., Dogan M. D., Patel S., Pétervári E., Balaskó M., Wanner S. P., Eales J., Oliveira D. L., Gavva N. R., Almeida M. C., Székely M., and Romanovsky A. A. (2011) The hypothermic response to bacterial lipopolysaccharide critically depends on brain CB1, but not CB2 or TRPV1, receptors. J. Physiol. 589, 2415–2431 21486787 10.1113/jphysiol.2010.202465 PMC3098711

